# A Fully Automated System Using A Convolutional Neural Network to Predict Renal Allograft Rejection: Extra-validation with Giga-pixel Immunostained Slides

**DOI:** 10.1038/s41598-019-41479-5

**Published:** 2019-03-26

**Authors:** Young-Gon Kim, Gyuheon Choi, Heounjeong Go, Yongwon Cho, Hyunna Lee, A-Reum Lee, Beomhee Park, Namkug Kim

**Affiliations:** 1 Department of Biomedical Engineering, Asan Institute of Life Science, University of Ulsan College of Medicine, Asan Medical Center, 88 Olympic-ro 43-gil, Songpa-gu Seoul, South Korea; 2 Department of Convergence Medicine, University of Ulsan College of Medicine, Asan Medical Center, 88 Olympic-ro 43-gil, Songpa-gu Seoul, South Korea; 3 Center for Superintelligence, Seoul National University, 08826 Seoul, South Korea; 4 Department of Pathology, University of Ulsan College of Medicine, Asan Medical Center, 88 Olympic-ro 43-gil, Songpa-gu Seoul, South Korea

## Abstract

Pathologic diagnoses mainly depend on visual scoring by pathologists, a process that can be time-consuming, laborious, and susceptible to inter- and/or intra-observer variations. This study proposes a novel method to enhance pathologic scoring of renal allograft rejection. A fully automated system using a convolutional neural network (CNN) was developed to identify regions of interest (ROIs) and to detect C4d positive and negative peritubular capillaries (PTCs) in giga-pixel immunostained slides. The performance of faster R-CNN was evaluated using optimal parameters of the novel method to enlarge the size of labeled masks. Fifty and forty pixels of the enlarged size images showed the best performance in detecting C4d positive and negative PTCs, respectively. Additionally, the feasibility of deep-learning-assisted labeling as independent dataset to enhance detection in this model was evaluated. Based on these two CNN methods, a fully automated system for renal allograft rejection was developed. This system was highly reliable, efficient, and effective, making it applicable to real clinical workflow.

## Introduction

Convolutional neural networks (CNNs) are state-of-the-art machine learning techniques that have led to many breakthroughs in image classification^1,2^, object detection^3,4^, and segmentation^5,6^. Applications of CNN to medicine have improved the performance of computer aided diagnosis^7,8^. Deep learning performance of CNNs may be enhanced by using massive datasets. Although the use of massive manual labeled datasets is highly time-consuming, these datasets showed comparable performance to expert clinicians. For example, the diagnostic performance of a CNN model, trained using 130 K fundus images, was comparable to that of expert ophthalmologists in diagnosing diabetic retinopathy^7^. Moreover, a CNN model trained using 130 K dermoscopy images and patients’ skin images was as accurate as dermatologists in distinguishing skin carcinoma from benign lesions^8^.

CNNs can be used to develop fully automatic pathologic diagnosis systems. Although it is ideal to examine the entire area of a specimen with an light microscope, it is impossible to closely examine all specific regions of each specimen in real clinical settings. Thus, to reach a final diagnosis pathologists alter the magnification. Moreover, pathologic images are very complex, with eye fatigue reducing diagnostic accuracy over time. In addition, subjective evaluations might be susceptible to inter- and/or intra-observer variations. These drawbacks may be overcome by CNNs. For example, CNNs have been applied to giga-pixel immunostained images to detect breast cancer metastases to sentinel lymph nodes^9–11^ and prostate cancer in biopsy specimens^11,12^. CNN models have also been applied to immunostained images to detect brain and colon cancers^13,14^.

The demand for kidney transplants is increasing worldwide. Renal biopsy is the gold standard for the evaluation of allograft rejection. Deposition of C4d in peritubular capillaries (PTCs), the tiny blood vessels surrounding renal tubules, is an established marker of antibody-mediated allograft rejection^15^. C4d score, defined as the proportion of C4d positive PTCs on immunostaining^16^, is one of the most important factors in the diagnosis of antibody-mediated rejection. Ideally, C4d score should be determined by counting all C4d positive and negative PTCs. However, it is practically impossible for pathologists to quantify all PTC samples, as the microscopic evaluation of PTCs is too time consuming, poor reproducible, and labor-intensive. Pathologists must therefore visually estimate the proportion of C4d positive PTCs. However, they may overlook some microscopic foci or inaccurately estimate the proportion of C4d positive PTCs, and their estimates may be susceptible to inter- and/or intra-observer variations^17–19^. Because automated PTC counting may result in a more accurate diagnosis, deep learning studies using CNN models are required to diagnose allograft rejection in kidney transplant recipients.

**Figure 1:**
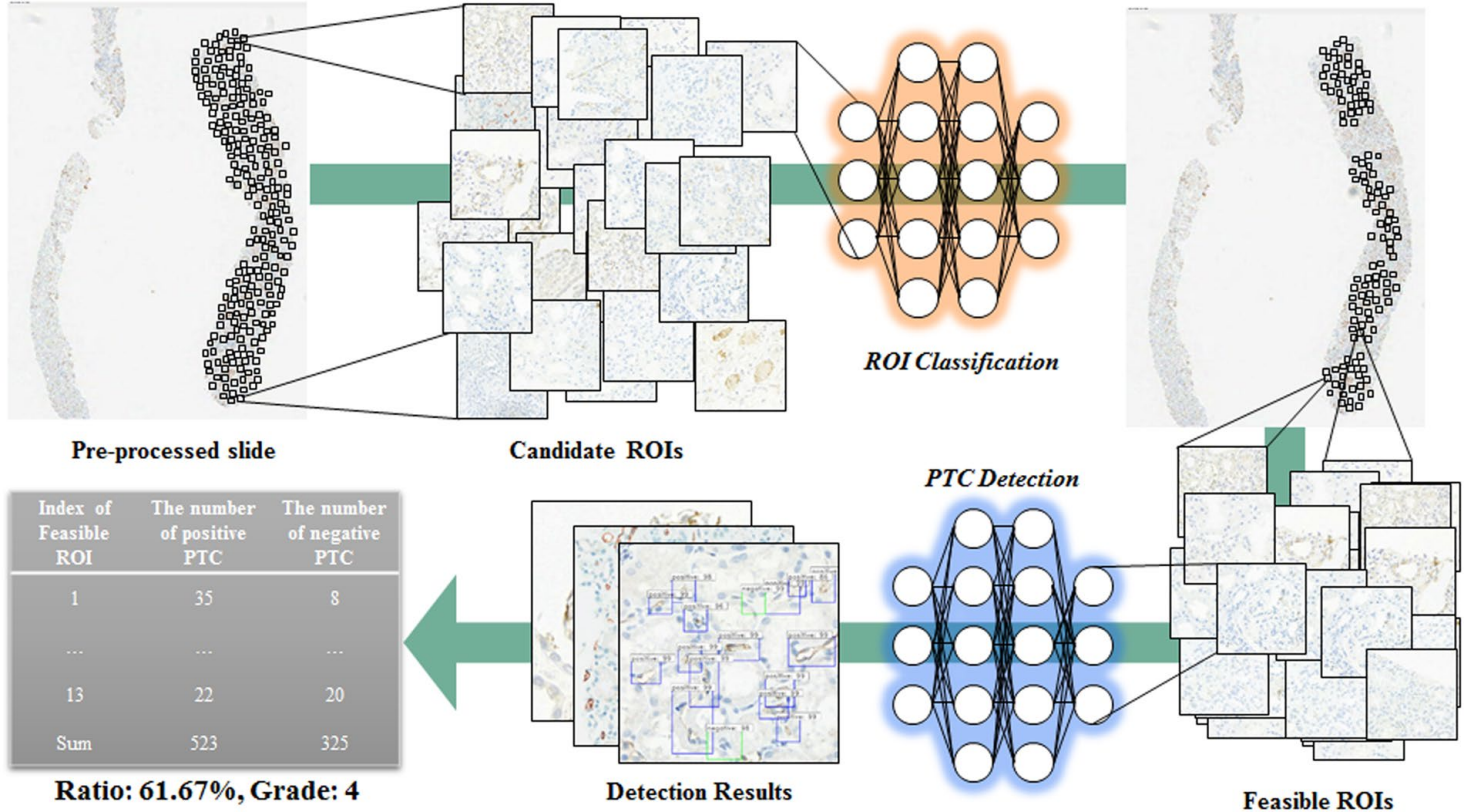
Overall procedure of our proposed method.

To develop clinically applicable system to identify regions of interest and to detect C4d positive and negative peritubular capillaries in giga-pixel immunostained slides, we proposed and evaluated deep-learning-assisted labeling with more efficiency, enhancing the detection model with pathologists’ insights with enlarged masks, and a fully automated system with combining CNN based classification and detection as routine pathologists’ workflow to predict renal allograft rejection. The overall procedure is described in Fig. 1. The system scans digital images from immunostained pathologic slides and removes background areas by Otsu’s thresholding^20^. Histogram equalization is processed to reduce variations such as illumination or degree of staining. After selecting all candidate regions of interest (ROIs) with sufficient tissue in each slide, the CNN model classifies ROIs as feasible or non-feasible and detects all C4d positive and negative PTCs in feasible ROIs to determine C4d scores. To train the CNN detection model, enlarged masks with certain sized margins are used as input, so that each enlarged mask includes neighborhood information, such as renal tubules, present near the PTCs. Deep-learning-assisted labeling from independent dataset are used for results determined by the detection model which was trained using dataset by manual labeling. The effectiveness of the enlarged mask and deep-learning-assisted labeling was assessed by comparison with FROC. The CNN detection model using enlarged masks trained with margin sizes of 50 and 40 pixels performed better than those without enlarged masks for the detection of C4d positive and negative PTCs, respectively. In comparisons of deep-learning-assisted labeling, the CNN detection model trained with either or both data by deep-learning-assisted labeling performed better than the model trained with data by manual labeling.

## Materials and Methods

The institutional review board for human investigations at Asan Medical Center (AMC) approved the study protocol with removal of all patient identifiers from the images, and waived the requirement for informed consent, in accordance with the retrospective design of this study. A total of 380 needle biopsies of renal allografts were obtained from patients who underwent renal transplantation at AMC from 2009 to 2016; all samples had been stored in the Department of Pathology.

To obtain representative samples, cases in the period were randomly selected without consideration for specific pathologic diagnosis. Consequently, 108 C4d positive and 272 C4d negative cases were retrieved including 46 zero-day allograft biopsies. Two pathologists meticulously reviewed all slides and modified false negative results; finally, 189 cases were classified as C4d positive and 191 cases as C4d negative. C4d was assessed immunohistochemically using a Ventana BenchMark XT autostainer (Ventana Medical Systems, Tucson, AZ, USA), and 380 whole slides were imaged using a digital slide scanner (Pannoramic 250 Flash, 3DHISTECH, Budapest, Hungary) with a 20× objective lens (specimen-level pixel size, 0.221 × 0.221 μm). All samples were anonymized before analysis and labeling.

The 380 slides were divided into subsets 1 and 2, consisting of 200 and 180 slides, respectively, to validate the feasibility of using deep-learning-assisted labeling. Subset 1 was used to train the CNN model for classification and detection, and subset 2 was used to validate the model. The slides in the two subsets were randomized for training (60%), test (20%), and validation (20%). Computational complexity of feasible ROI classification and PTC detection takes constant time. The average time (and standard deviation) for automatic system per slide was 785.81 (±176.97) sec. In this average time, the time for classification per slide and detection per ROI was 712.23 (±132.62) and 0.49 (±0.02) sec where the number of average feasible ROIs was 147.61 (±105.02).

### Feasible ROI Classification

**Figure 2:**
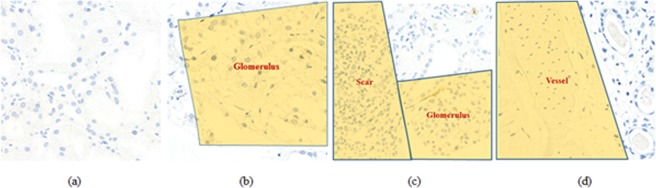
Decision criteria to classify feasible and non-feasible ROIs. (**a**) Feasible ROI, (**b**–**d**) non-feasible ROIs from dominant ambiguous regions including scar, glomerulus, and vessels.

All randomly identified candidate ROIs were independently labeled by three pathologists as feasible or non-feasible criteria. Sensitivity was maximized by identifying as many feasible ROIs as possible within each slide. A ROI was classified as non-feasible when more than two-thirds of its image consisted of suboptimal areas, defined as 1) an artifact or poorly stained area that limited proper interpretation; 2) areas without PTCs, such as a large vessel, glomerulus, or vacant area; and 3) scarred or infracted areas^21^. Examples for criteria are shown in Fig. 2.

A ROI size of 1024 × 1024 pixels was defined by the pathology team, as an image of this size provided a field of vision similar to a 400 × optical microscopic view, the maximum magnification used in routine practice. The total number of ROIs in subset 1 was 2723, including 2134 feasible and 769 non-feasible ROIs.

The CNN classification model was trained to classify feasible and non-feasible ROIs using an Inception v3 network^22^ and an ImageNet pre-trained model^23^. To prevent model overfitting from unbalanced data, adjacent regions were assessed to equalize proportions between classes. Augmentation methods in real time included horizontal and vertical flipping, rotation (0–90°), and zooming in and out (0–10%). The model was implemented in Keras using the Tensorflow with an NVIDIA GTX 1080 Ti GPU, binary-cross entropy loss, stochastic gradient descent optimizer (SGD) with learning rates of 10^−5^, and dropout with probability of 0.5. The learning rate was reduced to one-tenth per one-third of total epochs 2000 and more detailed parameters are listed in Table 1. Training was terminated at the lowest loss of the test set. The performance of the CNN classification model was evaluated by determining its sensitivity and specificity.

**Table 1:** Parameters used for training CNN classification model and CNN detection model.

Classification model		Detection model	
Optimizer	SGD	Optimizer	Adam
Learning rate	1e-5	Learning rate	1e-5
Weight decay	1e-6	Weight decay	0.0
Epochs	2000	Epochs	150
Momentum	0.9	*β*1, *β*2	0.9, 0.999
		Epsilon	1e-4

### PTC Detection

Three pathologists independently labeled C4d positive and C4d negative PTCs in feasible ROIs of subset 1 by hand drawing using in-house software. After completing these tasks by self, they had a meeting for the discussion of conflicted cases and made a consensus. In addition, pseudo negative PTCs, consisting of non-PTC regions, such as tubules and glomeruli that can be confused with PTCs, were drawn to train the model robustly.

**Figure 3:**
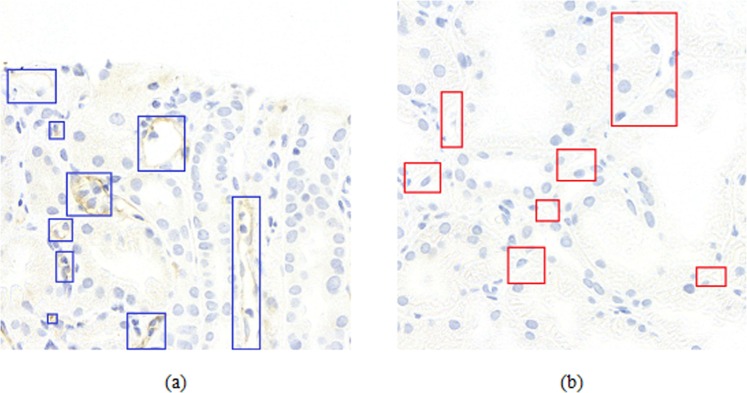
Gold standard examples of C4d negative and positive in PTC. Blue and red rectangles show the positive and negative PTC in (**a**) and (**b**), respectively.

Widths and heights of labeled PTC masks ranged from 25 to 392 pixels. A total of 1823 PTCs were identified by manual labeling, including 549 C4d positive and 1274 C4d negative PTCs, whereas a total of 3836 PTCs were identified from data by deep-learning-assisted labeling, including 1597 C4d positive and 2239 C4d negative PTCs. Examples are shown in Fig. 3.

**Figure 4:**
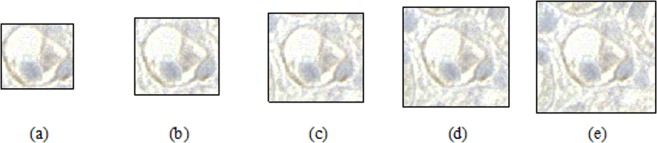
Example of labeled C4d positive PTC with various margin sizes. Margin sizes of (**a**) 0, (**b**) 10, (**c**) 20, (**d**) 30, (**e**) 40 pixels.

In general object detection tasks, the object is normally placed on a complex or object-independent background, with object boundaries determined using fitted coordinates. However, pathologic detection of PTCs is different. Because PTCs are capillaries located near tubules, their presence constitutes additional information during training using an enlarged rather than a fitted mask as shown in Fig. 4. An enlarged mask was used because the boundaries of tubules near PTCs could help recognize PTCs on slides. To evaluate the optimal enlarged margin size around manual labeled data, various margin sizes (0–80 pixels at 10-pixel intervals) were adjusted when training the detection model.

**Figure 5:**
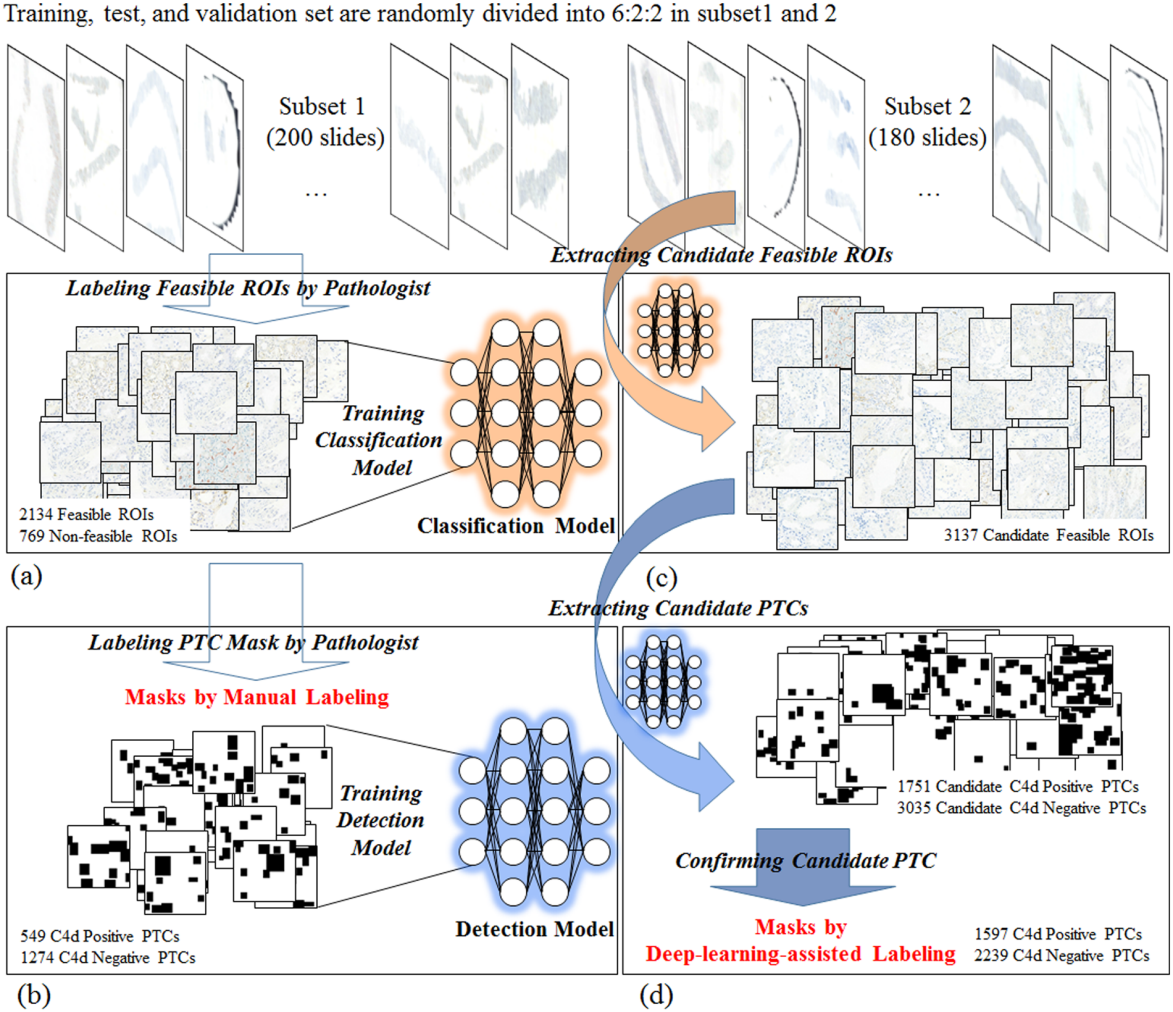
Sequence for deep-learning-assisted labeling. All slides are randomly divided into 6:2:2 as training, test, and validation set in subset 1 and 2. (**a**) Training classification model with feasible ROIs in subset 1. (**b**) Training detection model with manual labeled masks in the feasible ROIs (**c**). Extracting candidate feasible ROIs in subset 2 by the classification model. (**c**) Extracting candidate PTCs by the detection model and confirming results of (**d**) as deep-learning-assisted labeling.

Labeling for massive amounts of data to enhance deep learning performance is time- and labor-intensive. These drawbacks may be overcome by using data by deep-learning-assisted labeling in place of or in addition to manual labeled data. A deep learning model trained for detection can be used to evaluate candidate objects first, followed by confirmation or modification involving little labor to acquire massive data, with the latter called data by deep-learning-assisted labeling. Deep-learning-assisted labeling can reduce the labor required. Figure 5 shows a process used to acquire deep-learning-assisted labeling for detection of C4d positive and negative PTCs. Firstly, two types of CNN model were trained from subset 1 with feasible ROIs and manual labeled mask data (Fig. 5(a,b)). The CNN classification model trained from subset 1 was used to identify feasible ROIs in subset 2 (Fig. 5(c)), and the CNN detection model trained from subset 1 was used to identify candidate C4d positive and negative PTCs in all feasible ROIs. Finally, data by deep-learning-assisted labeling were selected by confirming all candidate PTCs as being C4d positive or C4d negative using an in-house re-labeling tool (Fig. 5(d)). In addition, this procedure was used to test false negative PTCs not detected by the model. If the center of the boundary box identified by the detection model did not deviate significantly from the center of the actual PTC, the PTC was confirmed as C4d positive or negative.

C4d negative and positive PTCs were detected using region proposal based Faster R-CNN detection algorithm^4^ with ImageNet pre-trained model based Resnet50^6^. Augmentation methods in real time included horizontal and vertical flipping, rotation (0–90°), and zooming in and out (0–10%). The model was implemented in Keras using the Tensorflow with an NVIDIA GTX 1080 Ti GPU. Smooth L1 loss for bounding box regression and categorical-cross entropy loss for classification network in backbone were used. Adam optimizers with learning rates of 10^−5^ for region proposal and classification network. More detailed parameters are listed in Table 1. Training was terminated at the lowest loss of the test set. Training was terminated at the lowest loss of the test set.

To evaluate the effectiveness of enlarging margins with faster R-CNN detection algorithm and of using data by deep-learning-assisted labeling with faster R-CNN and one-shot based YOLO v2 detection algorithm^24^, FROC scores, defined as the average sensitivity at seven predefined false positive rates (1/8, 1/4, 1/2, 1, 2, 4, and 8) per ROI, were calculated.

Stress test to see if the 380 slides datasets where the number of positive PTC and negative PTC masks were 2146 and 3513 are sufficient was conducted. To train the different detection CNN model performance with Faster R-CNN for detecting C4d positive and negative PTCs with different amount of training data, all labeled data including subset 1 and subset 2 were used. All data were shuffled and divided into 80% and 20% as training and fixed validation set. Of training set, different training data were randomly selected to train each model at rates of 40%, 60%, 80%, and 100%. Test set for tuning each detection models were randomly selected at rages of 10% in each different training data. To measure performance for detecting C4d positive and negative PTCs, relative sensitivities at as sensitivity were calculated.

## Results

### Feasible ROI Classification

The CNN classification model trained from subset 1 was tuned with high specificity to minimize false positives. The sensitivity and specificity of the CNN classification model were 0.7951 and 0.9941, respectively.

**Figure 6:**
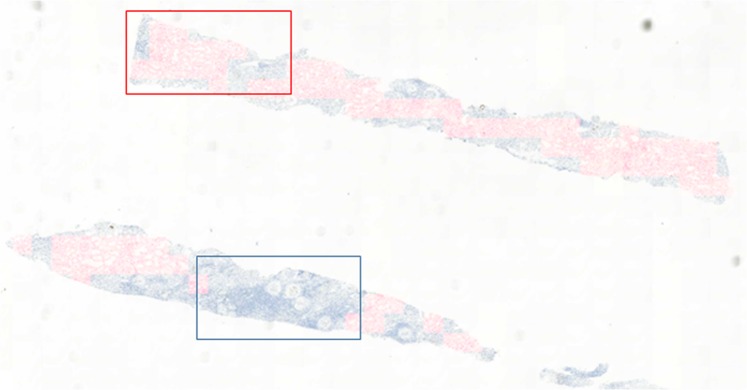
Feasible and non-feasible ROI classification results. Tissues including feasible ROIs are colored red.

To validate the use of deep-learning-assisted labeling, the CNN classification model trained from subset 1 was used to determine candidate feasible ROIs in subset 2. This model was used to extract feasible ROIs from all tissue regions of subset 2 with high specificity. The mean ± SD number of ROIs per slide was 89.23 ± 34.22. An example of classification results for all regions of a slide is shown in Fig. 6. Tissues containing feasible and non-feasible ROIs were colored red. ROIs containing tubules with PTCs were classified as feasible, whereas ROIs containing scars and glomeruli were classified as non-feasible.

### PTC detection

**Figure 7:**
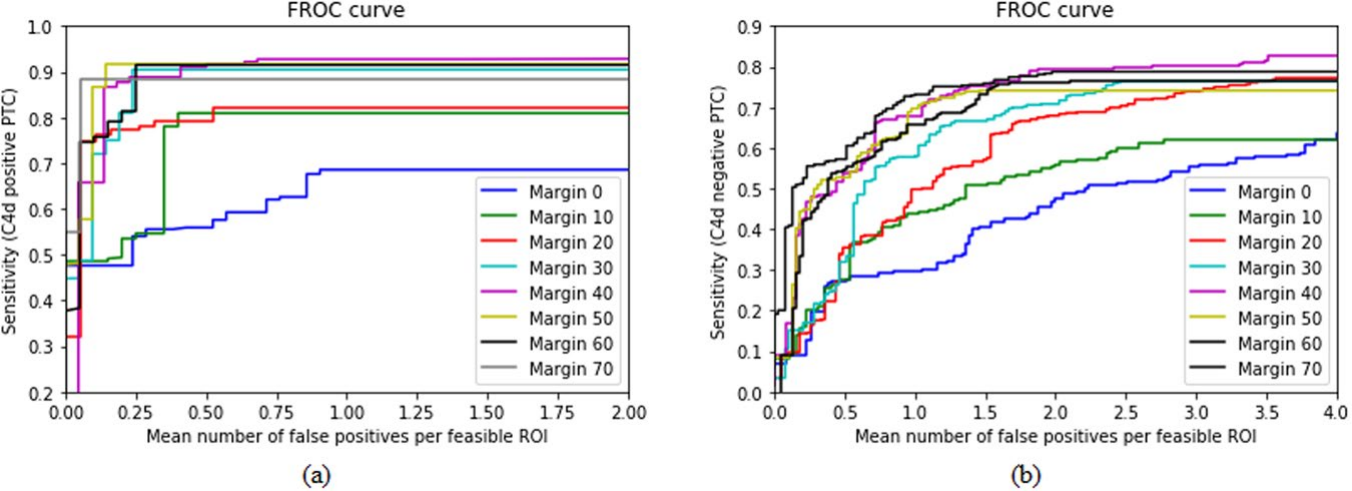
FROC comparisons at different size of margin on manual labeled data. Results for detection of (**a**) C4d positive and (**b**) negative PTC.

The performances of validations using margin sizes of 0–70 pixels to detect C4d positive and negative PTCs on manual labeled data were compared in Fig. 7 and Table 2 with Faster R-CNN detection algorithm. FROC scores and overall sensitivities for the detection of C4d positive and negative PTCs increased as margin sizes increased. However, overall sensitivities and FROC scores in detecting C4d positive PTCs were optimal at margin sizes of 50 pixels, decreasing at 60 pixels (Fig. 7(a)). Similarly, overall sensitivities and FROC scores in detecting C4d negative PTCs scores were optimal at 40 pixels (Fig. 7(b)). FROC scores were highest for models trained with margin sizes of 50 and 40 pixels for the detection of C4d positive and negative PTCs, respectively.

**Table 2:** The sensitivities and FROC scores for faster R-CNN detection of C4d positive and negative PTC with various margin sizes (0 to 70) at different mean number of false positives per feasible ROI.

Mean of FPs	Margin size for detection of C4d positive PTC							
	0	10	20	30	40	50	60	70
0.125	0.4767	0.4862	0.7627	0.7200	0.6575	0.8667	0.7571	0.8851
0.250	0.5397	0.5345	0.7740	0.9045	0.8883	0.9167	0.8136	0.8851
0.500	0.5587	0.8092	0.7910	0.9045	0.9106	0.9167	0.9148	0.8851
1.000	0.6854	0.8092	0.8192	0.9045	0.9274	0.9167	0.9148	0.8851
2.000	0.6854	0.8092	0.8192	0.9045	0.9385	0.9167	0.9148	0.8851
4.000	0.6854	0.8092	0.8192	0.9045	0.9385	0.9167	0.9148	0.8851
8.000	0.6854	0.8092	0.8192	0.9045	0.9385	0.9167	0.9148	0.8851
Score	0.6166	0.7238	0.8006	0.8781	0.8856	**0.9095**	0.8778	0.8851
0.125	0.0919	0.0935	0.0976	0.1519	0.2306	0.0944	0.0918	0.4087
0.250	0.1258	0.2019	0.1453	0.1706	0.4663	0.4485	0.4248	0.5549
0.500	0.2733	0.2783	0.3541	0.3200	0.5388	0.5272	0.5465	0.5720
1.000	0.2952	0.4367	0.4978	0.5774	0.6789	0.6969	0.6571	0.7293
2.000	0.4655	0.5553	0.6792	0.7080	0.7920	0.7412	0.7611	0.7876
4.000	0.6217	0.6195	0.7743	0.7633	0.8274	0.7412	0.7633	0.7876
8.000	0.7257	0.6195	0.7743	0.7655	0.9004	0.7412	0.7633	0.7876
**Score**	0.3713	0.4006	0.4746	0.4938	**0.6334**	0.5700	0.5725	0.6611

**Figure 8:**
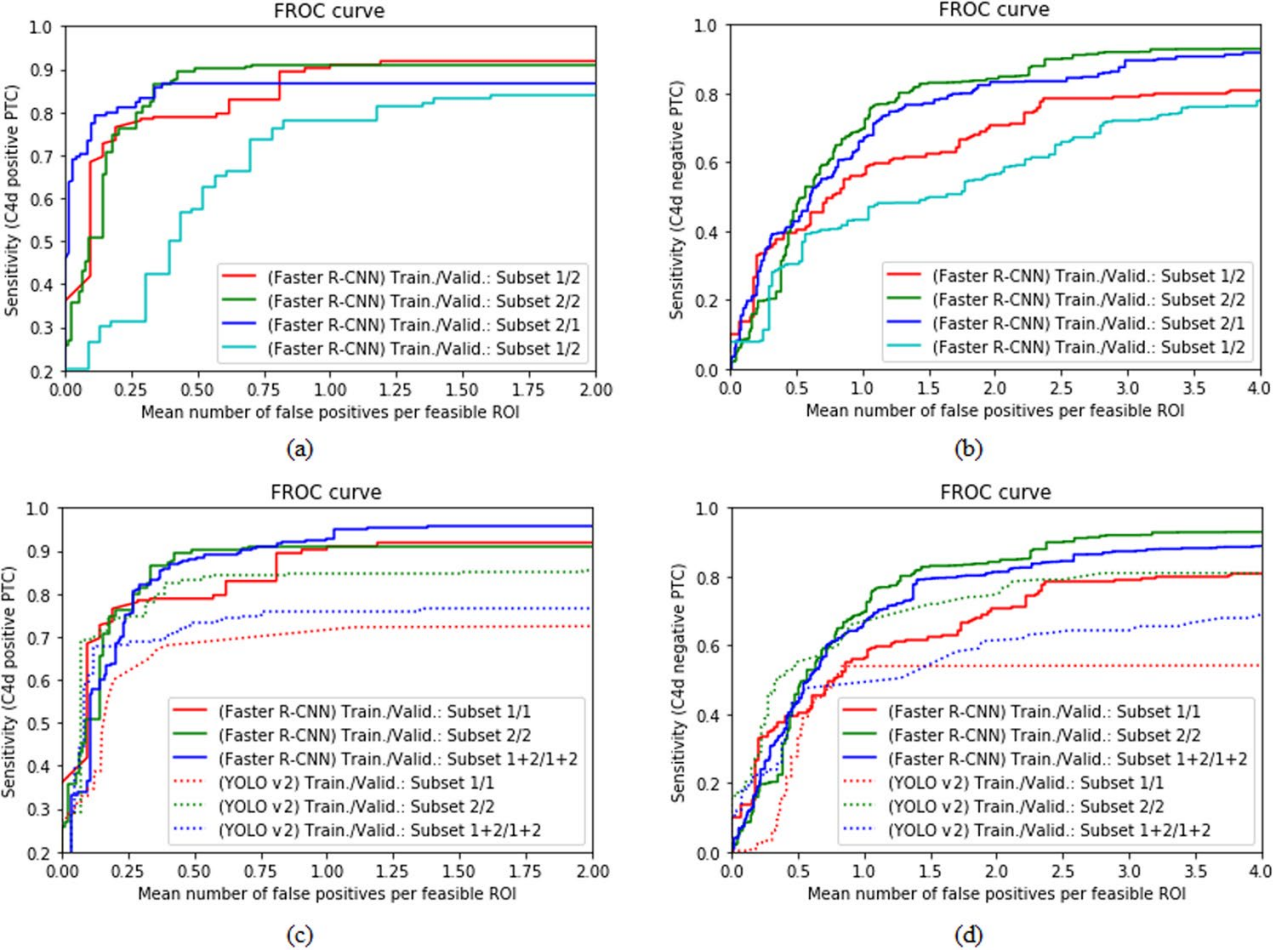
FROC comparisons for validation of feasibility of using deep-learning-assisted labeling. FROC comparisons to show inter- and intra-observer variation between different validation set for detection of (**a**) C4d positive and (**b**) negative PTC with faster R-CNN detection algorithm. FROC comparisons to validate effectiveness of deep-learning-assisted labeling for detection of (**c**) C4d positive and (**d**) negative PTC with faster R-CNN and YOLO v2 detection algorithms.

The CNN detection models trained with margins of 40 pixels for the detection of C4d positive and negative PTCs were tuned to maximum sensitivity to generate as much data by deep-learning-assisted labeling as possible. Deep-learning-assisted candidate labeled data were generated by the CNN detection models, which have a recall and precision of 0.8821 and 0.9384, respectively, for the detection of C4d positive PTCs, and of 0.8094 and 0.7108, respectively, for the detection of C4d negative PTCs. The characteristics of manual and deep-learning-assisted labeling differed slightly, in that manual labeled data only included masks fitted to both classes, whereas deep-learning-assisted labeling also included masks that were slightly misplaced locally. Figure 8(a,b) shows inter- and intra-observer variations, respectively, between subset 1 (manual labeling) and subset 2 (deep-learning-assisted labeling). To validate the feasibility of using deep-learning-assisted labeling, FROC scores and sensitivities were compared in models trained with data by manual labeled, data by deep-learning-assisted labeling, and both together at different mean numbers of false positive PTCs per feasible ROI with two different type of detection algorithm. In detecting C4d positive and negative PTCs, the Faster R-CNN model showed better accuracies than those of YOLO v2 model. In addition, both models trained by subset 2 or fusion dataset including subset 1 and subset 2 showed better accuracies (Fig. 8(c,d) and Table 3).

**Table 3:** The sensitivities and FROC scores for faster R-CNN and YOLO v2 detections of C4d positive and negative PTC with different detection models trained by different dataset at different mean number of false positives per feasible ROIs (0 to 2 and 0 to 8 for detection of positive and negative PTC, respectively). Model 1: trained by subset 1, Model 2: trained by subset 2, Model 3: trained by fusion of subset 1 and 2.

Mean of FPs	Detection model for C4d positive PTC			Detection model for C4d negative PTC		
	Model 1	Model 2	Model 3	Model 1	Model 2	Model 3
**Faster R-CNN**						
0.125	0.6970	0.5495	0.5768	0.1387	0.0863	0.1148
0.250	0.7803	0.6923	0.7510	0.3333	0.1969	0.2405
0.500	0.7886	0.8791	0.8817	0.3966	0.4579	0.4343
1.000	0.9024	0.9451	0.9253	0.5615	0.6969	0.6644
2.000	0.9187	0.9478	0.9585	0.7082	0.8424	0.8131
4.000	0.9187	0.9478	0.9647	0.8075	0.9294	0.8887
8.000	0.9187	0.9478	0.9647	0.8563	0.9294	0.8910
**Score**	0.8463	0.8442	**0.8603**	0.5431	**0.5913**	0.5781
**YOLO v2**						
0.125	0.3864	0.7009	0.6736	0.0058	0.2034	0.1928
0.250	0.6284	0.7479	0.6795	0.0032	0.3644	0.2240
0.500	0.6817	0.8333	0.7329	0.2945	0.5512	0.4494
1.000	0.7124	0.8462	0.7567	0.5394	0.6617	0.4761
2.000	0.7221	0.8547	0.7565	0.5423	0.7480	0.6121
4.000	0.7444	0. 8761	0.7864	0.5423	0.8100	0.7106
8.000	0.7444	0.8846	0.7864	0.5423	0.8100	0.7345
**Score**	0.6599	**0.8112**	0.7388	0.3528	**0.5926**	0.4856

**Figure 9:**
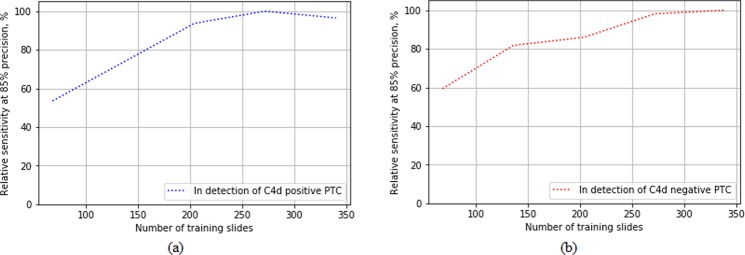
Relative sensitivities comparisons of detection models trained with different amount of training data for detecting (**a**) C4d positive and (**b**) negative PTC with faster R-CNN detection algorithm.

The CNN detection models for detecting C4d positive and negative PTCs trained with different amount of training dataset were compared as shown in Fig. 9. The performances in detection of C4d positive and negative PTC were shown to be saturated at around 300 slides.

## Discussion

To develop clinically applicable system, deep-learning-assisted labeling with more efficiency, enhancing the detection model with pathologists’ insights, combining CNN based classification and detection for a fully automated system were developed in this paper. Training the CNN detection models with enlarged masks surrounding PTC region is inspired by the actual pathologists’ insights, which enhanced the detection performance highly (Fig. 7 and Table 2). Deep learning models generally need massive data for training. To overcome the problem of small dataset, we tried to determine a feasibility of deep-learning-assisted labeling that is made from independent dataset with low-labor, which could alleviate massive manual labor. In our experiments, using deep-learning-assisted labeling for training, the performance of the detection model was enhanced (Fig. 8(c,d) and Table 3) compared with only dataset by using manual labeling only, because deep-learning-assisted labeling help draw PTC masks more robustly with less variations such as inter- and/or intra-observer, illumination, and degree of staining. In addition, massive dataset would improve the performance of the CNN detection models. In the stress test, we showed that 380 slides were sufficient to train CNN detection model for finding positive and negative PTC.

Deep learning on pathologic images could depend on various scanning conditions such as not only illumination, but also the degree of staining, different equipment, and so on. To overcome these problems, pre-trained network which has been already trained with bunch of tremendous number of images having a variety of complex variation, histogram normalization that is one of stain normalization method, and doubled dataset by deep-learning-assisted labeling were used to train our deep learning model to have robustness of them.

The most important aspects of application of this system to pathology are full automation for objective diagnosis and alleviation of manual labor. This study proposed a fully automated two-step CNN system for the diagnosis of allograft rejection. The first step consists of the use of a CNN classification model to identify feasible ROIs in all tissue regions and the second step consists of the use of a CNN detection model to identify and count C4d positive and negative PTCs, a marker of allograft rejection in kidney transplant recipients. These findings suggest that this system may be applicable to most tasks in digital pathology.

Classification of all tissue regions as feasible or non-feasible ROIs using the CNN classification model is practical, as pathologists cannot determine all feasible ROIs in a tissue sample and have difficulty identifying negative PTCs. By contrast, the CNN classification model can precisely evaluate the entire specimen, and the CNN detection model can accurately count the numbers of C4d positive and negative PTCs in all feasible ROIs. Determining both C4d positive and C4d negative PTCs may alter clinical diagnoses.

In addition, two kinds of performance comparisons were conducted. Firstly, the performances of models trained with different size of margin including PTC region were compared. Enlarged mask with a certain size improved detection CNN model, which method was mimicked by a real clinical experience. This novelty including surrounding regions could be used widely for similar tasks. Secondly, the performances of models trained with data by manual labeling and data by deep-learning-assisted labeling were compared. The generation of data by deep-learning-assisted labeling and confirmation by expert pathologists may help improve the performance of these models. Pathologic labeling is very difficult, even for expert pathologists, whereas the deep-learning-assisted method generated relatively robust labels.

Several obstacles should be overcome before clinical application. The sample size (380 slides) is about 1.2 times the average renal allograft biopsy per year in this center, which is one of the largest medical center in South Korea. Though it is also relatively larger than other studies related to pathologic assessment using convolutional neural network^9,11,12^, we will try to evaluate the performance of this more with wild dataset from larger data. Also, all cases were recruited from a single center using only one slide scanner, which could lead to less variations such as background illumination or degree of staining. To evaluate the robustness of this method, further studies with multi-center could be needed. In addition, comparisons of the performance and outcomes of this method with those of pathologists are needed to determine the clinical effectiveness of this system.

## Conclusion

Pathologic examination is time-consuming, involving the examination of all areas of cells using a digital microscope. Subjectively determined pathologic diagnoses may differ and may be easily susceptible to inter- and/or intra-observer variations. These drawbacks may be overcome by an automatic method of PTC scoring using two types of trained models (window classification and PTC detection). Classification, detection, and scoring comparisons showed that this method yielded reasonable results when evaluating stained giga-pixel digital slides. Use of this system may be feasible diagnostically in detecting other diseases and conditions.
